# Can Blood-Circulating Factors Unveil and Delay Your Biological Aging?

**DOI:** 10.3390/biomedicines8120615

**Published:** 2020-12-15

**Authors:** Natalia Rybtsova, Tatiana Berezina, Alexander Kagansky, Stanislav Rybtsov

**Affiliations:** 1 Centre for Regenerative Medicine, Institute for Regeneration and Repair, University of Edinburgh, Edinburgh EH16 4UU, UK; rybnat@yahoo.com; 2 Department of Scientific Basis of Extreme Psychology, Moscow State University of Psychology and Education, 127051 Moscow, Russia; tanberez@list.ru; 3 Centre for Genomic and Regenerative Medicine, School of Biomedicine, Far Eastern Federal University, 690922 Vladivostok, Russia

**Keywords:** aging, metabolic disorders, blood factors, inflammation, senescence, aging biomarkers, biological age

## Abstract

According to the World Health Organization, the population of over 60 will double in the next 30 years in the developed countries, which will enforce a further raise of the retirement age and increase the burden on the healthcare system. Therefore, there is an acute issue of maintaining health and prolonging active working longevity, as well as implementation of early monitoring and prevention of premature aging and age-related disorders to avoid early disability. Traditional indicators of biological age are not always informative and often require extensive and expensive analysis. The study of blood factors is a simple and easily accessible way to assess individual health and supplement the traditional indicators of a person’s biological age with new objective criteria. With age, the processes of growth and development, tissue regeneration and repair decline; they are gradually replaced by enhanced catabolism, inflammatory cell activity, and insulin resistance. The number of senescent cells supporting the inflammatory loop rises; cellular clearance by autophagy and mitophagy slows down, resulting in mitochondrial and cellular damage and dysfunction. Monitoring of circulated blood factors not only reflects these processes, but also allows suggesting medical intervention to prevent or decelerate the development of age-related diseases. We review the age-related blood factors discussed in recent publications, as well as approaches to slowing aging for healthy and active longevity.

## 1. Introduction

The individual biological age can differ significantly from the calendar age due to the individual’s lifestyle and stochastic influence of environmental factors, as well as genetic and epigenetic factors that determine ontogenesis [1]. The biological age is usually estimated using morphological, physiological, and functional characteristics of the organism and is compared with the average biological age of other individuals of the same calendar age [2,3,4,5]. In particular, the study of the state of the vascular system is often used to assess the biological age [6]. The rate of aging, which determines the biological age, also depends on various factors, including the diet and bad habits [7,8]. Individual aging can accelerate due to stress associated with social upheaval and personal problems [2,9]. Assessment of individual biological age and aging markers’ identification are necessary to predict life expectancy, to evaluate the risk of developing age-related diseases, and to work out the measurable parameters of geroprotection for the rapidly expanding anti-aging research [10,11,12,13,14].

This is especially important for extending the employment age and active longevity in the modern society in the context of the gradual increase of the retirement age across both the developed and developing countries. Thus, the analysis of big data, including various genetic, metabolic, and medical indicators associated with biological aging, demonstrated that dietary restrictions [15,16], weight loss, exercise [17,18], and even an improvement of the intestinal microbiota [19,20,21,22] can reverse the rise in markers of aging and prevent pathological conditions, including obesity, diabetes, cancers, etc., showing great promise for increasing the net average active life expectancy in the global population [23,24].

Aging is a major potential risk factor for a vast spectrum of conditions such as immunodeficiency, cardiovascular disease, diabetes, cancer, and various neurological disorders. There are no effective treatments for many of these diseases, and aging reduces the effectiveness of treatments despite significant efforts in research and clinical investment over several decades [13,25,26].

There are two approaches we believe can extend active healthy life: preventing premature aging and preventing the development of senile diseases. Understanding the causes of aging and age-related diseases is essential for implementing these strategies. Aging is a complex process of gradual degenerative changes in all body tissues. After birth, during our growth and development, new structures and tissues of the body are formed. In adulthood, such de novo formation and growth stop, gradually shifting towards maintaining the functions of the body and the balance of various systems. With age, the imbalance of the body systems increases, which leads to loss of control over organs and tissues, catabolism and wilting [27].

Although the complete genetic program of aging has not yet been discovered, scientists have identified many genes and processes that influence aging and lifespan [10,14,27,28,29,30]. One of the main mechanisms of aging is the accumulation of senescent cells in all organs and tissues [25,31]. Inflammation caused by infections or a predominance of inflammatory intestinal microbiota can exhaust the immune system [19,20,32]. Elevated levels of pro-inflammatory cytokines cause local tissue damage. The repair of damaged cells and tissues is an extremely energy- and resource- demanding process [33]. Growth factor and hormone signals significantly activate lipid metabolism, protein synthesis and proliferation, therefore, the maintenance of “order” in cells is forced off. Autophagy and mitophagy are suppressed, the secretion of enzymes that remove free radicals decreases. All this, in total, leads to the accumulation of damaged mitochondria and temporary cell dysfunction [33,34]. After regeneration, the cell cleaning and maintenance restart, and the cell functioning returns to normal. This is especially characteristic of stem cells, which are responsible for regeneration and restoration of damaged tissue during expansion and differentiation [35]. But in chronic inflammation, instead of cleansing (autophagy and mitophagy), cells are prone to senescence [36]. These senescent cells with increased metabolism acquire the so-called senescence-associated secretory phenotype: they begin releasing a large panel of pro-inflammatory factors [37]. A pathological loop arises—inflammation increases with age and, therefore, the number of senescent or exhausted cells grows. Regeneration is blocked due to the accelerated aging or/and depletion of regenerative stem cells. The increased inflammatory response and the accumulation of senescent cells are interdependent processes often called “senoinflammation” [25,38]. The accuracy of this concept is confirmed by the successful deceleration of age-related changes when using senolytic drugs that eliminate senescent cells [39,40,41,42] and anti-inflammatory therapy [43,44].

Inflammatory aging also affects hematopoietic stem and progenitor cells (HSPCs) [45,46,47,48,49]. It is characterized by age-related disorders of hematopoiesis and immunity, a decreased number of young cells and an increased amount of senescent cells in the circulation, reduction in phagocytic cells capable of cleansing tissues from accumulated transformed and old cells. Such aging of the immune system reduces immune surveillance, which leads to the emergence of foci of chronic infections, as well as of malignant proliferative disorders [50]. Hematopoietic cells not only carry out the immune function of cleansing tissues from aging cells and from infection, but also secrete a huge amount of pro- and anti-inflammatory and regenerative factors into the circulating blood [51]. The blood delivers nutrients, oxygen, hormones, and growth factors to all organs and tissues. Moreover, it also transmits orders to the entire body and controls the immune system [52]. An age-related change in the profiles of blood circulation factors reflects the processes and mechanisms of aging. The medical implementation of these factors into the bloodstream allows conveying signals to the internal body systems that regulate and monitor aging [27].

In this article, we present an overview of the latest research on the most significant reporters of aging. These are metabolic, hormonal, and inflammatory factors which provide objective criteria of biological age. Moreover, some of these factors directly affect the process of aging. In this review, we will focus only on the factors that can be measured in blood plasma.

## 2. Age-Related Changes in Plasma Biochemistry and Vascular Dynamics

Numerous studies of blood flow velocity under relaxation conditions have not revealed its significant decrease with age, either in animal or in human experiments. However, under stress, younger arteries dilate considerably compared to older ones. In addition, in older animals, the number of sensory neurons that control vascular tone and contraction has significantly decreased [53]. The number of capillaries reduces with age, their cross-section changes, and the likelihood of their local thrombosis increases [54]. Apparently, an increase in the fibrinogen amount affects blood clotting, even with a slight decline in the number of platelets in the elderly [55,56]. The elevation of the fibrinogen concentration in blood plasma with age reflects both the escalation of systemic inflammation and age-related changes in the vascular endothelium (Table 1) [57].

The risk of hasty organism dysfunctions increases with age. For example, the ratio of blood urea nitrogen to creatinine (BUN/creatinine), which is traditionally used in the clinical practice, also correlates with acute heart failure [58]. Another reliable marker of aging that gradually increases with age is the albumin/creatinine ratio, known as urine microalbumin. A very high level of microalbumin correlates with diabetes and hypertension that may trigger renal malfunction and failure [59].

It is widely known that the blood calcium level also increases with age, especially when associated with renal dysfunction. It has also been shown that at old age, a sudden drop or rise of calcium levels can cause a higher risk of premature death [57,60].

Blood cell parameters decreasing with age, such as the total lymphocyte count, red blood cell count, hemoglobin, and hematocrit, are routinely used to assess health [56]. It is well known that the hemoglobin level decreases between the ages of 50 and 90 in both men (from ~15.5 to 12.3 g/dL) and women (from ~13.5 to 11.5 g/dL) [61]. However, this correlation rather describes the increase in the incidence of pathological anemia with age; there are healthy aging individuals with stable hemoglobin levels [57].

New methods of analysis have made it possible to identify a wide range of additional components of blood plasma that change with age. Their role in aging and the possibility of using them for assessing biological age remain to be elucidated.

### 2.1. Lipids

The total amount of lipids and their variety (lipidome) are attenuated with age and can be considered age-related predictive markers of healthy lipid metabolism. Mass spectrometry analysis of the lipidome in blood plasma demonstrates the age-related increase in the concentration of some specific lipids regardless of the body mass index; they include cholesterol, sphingomyelins, and docosahexaenoic acid-containing phospholipids [62]. Age-dependent increase in both different classes of cholesterol and the inflammatory factors concentration correlates with the risk of cardiovascular disorders and life span reduction [63,64,65]. The triacylglycerol-containing polyunsaturated fatty acid (PUFA) accumulation in plasma during inflammation and aging correlates negatively with lifespan, while sphingomyelin concentration in plasma positively correlates with lifespan [66].

A recent report identified a group of biomarkers associated with the risk of a combination of age-related diseases. Metabolic profile monitoring of 44,168 people identified 14 biomarkers that correlate with all-cause mortality over 10 years. They include lipoproteins, fatty acids and glycolysis metabolites, markers of fluid balance, and inflammatory factors [67]. Another study revealed the genetic markers associated with plasma concentration of entire classes of lipids and the lipid modifications correlated with the risk of cardiovascular disorders [68].

### 2.2. NAD+/NADH Index

An age-dependent decrease in the level of NAD+ and its precursors causes a gradual mitochondrial dysfunction and an accumulation of metabolic disorders. The constitutive level of NAD+ depends on the balance between the NAD synthesis and the activity of the NAD+-consuming enzymes. NAD+ is essential for the immune function of hematopoietic cells during inflammation. All processes associated with aging, including inflammation, ischemia, metabolic imbalance, degenerative cell conditions, inhibit the NAD+ production; therefore, the concentration of NAD+ decreases twice every 20 years of human life [69,70]. NAD+ production is also diet-dependent. High protein intake leads to a decrease in the plasma NAD+ level [71]. Recent research discovered CD38-NADase as the main consumer of NAD+ and an agent responsible for the age-related NAD+ decline [72]. CD38 is expressed on a large subset of immune cells including B cells, T cells, NK cells, and some myeloid cells. Besides, CD38 abundantly is expressed on a large cohort of lymphoid leukemia cells. Given the importance of the NAD+ function in all metabolic processes, animal experiments have shown that blocking CD38-dependent NAD+ intake through chemical inhibition improves the health and lifespan of mice [73].

### 2.3. ROS

Reactive oxygen species (ROS) cause oxidative cell damage. ROS concertation increases with age; lowering ROS levels reduce age-dependent functional decline. For example, a methodology has recently been developed for assessing the amount of ROS by the number of their derivatives—reactive oxygen metabolites (D-ROM), as well as by the concentration of thiol protein groups indicating the state of the redox recovery system of the total thiol levels (TTL). This technique allowed finding a correlation between age-related changes and the accumulation of D-ROM and TTL markers. The D-ROM level and TTL concentration were also associated with the rate of premature death from cardiovascular diseases, oncological diseases, and diabetes [74,75]. 

The mechanism of redox-based balancing is also based on the transition of S-sulfenylated proteins Cys-SSH to Cys-SSOH (cysteine perthiosulfenic acid, oxidized form). Cys-SSH is broadly recognized as a cellular redox sensor. Sulfenylated cysteines of proteins (Cys-SSH) can be reversibly oxidated by ROS and reduce the free radicals’ level [76,77].

Genetic control over the elimination of free radicals and other toxins declines with age due to a decrease in *Nrf2* expression (nuclear factor erythroid 2 p45-derived factor 2) [78]. In turn, *Nrf2* controls expression of antioxidant proteins, detoxifying enzymes, drug transporters, and numerous cytoprotective proteins. Besides, *Nrf2* directly masters superoxide dismutase (SOD) expression through a responsive enhancer (SOD is an essential neutralizer of ROS) [78,79,80]. *Nrf2* can also protect mitochondria by directly regulating antioxidant enzyme function, increase the level of autophagy by upregulating Sirtuin (SIRT), and reduce inflammation by inhibiting NF-kB [81].

### 2.4. H_2_S

Hydrogen sulfide (H_2_S), soluble in blood plasm, also plays a fundamental role as a redox sensor in biological systems [82]. The discovery of the H_2_S-producing CSE (cystathionine γ-lyase) demonstrated that this molecule is not exclusively an environmental toxin. This helped to understand the role of H_2_S in the management of homeostasis, immune cells, tissue and organ function [83]. In the experiments on invertebrates, genetic deletion of the hydrogen sulfide/cysteine persulfide (H_2_S/Cys-SSH) producers shortened the lifespan of animals while boosting of H_2_S significantly increased it. An age-dependent decrease of the H_2_S concentration in plasma can be an objective indicator of age-related changes [82,84,85]. H_2_S and described above Cys-SSH can be easily measured by fluorescence and Dimedone switch tag methods [82,86].

Recently, it has been shown that H_2_S is involved in the regulation of the chaperone function. Enzymes controlling the H_2_S production CSE/CTH/CGL (cystathionine-γ lyase), CBS (cystathionine-β-synthase), and MST (3-mercaptopyruvate sulfurtransferase) are implicated in stress resistance, aging, and stress-dependent regulation of HSP22, HSP70, and HSF1 [87,88]. Chaperones play an important role in adaptive stress and can directly regulate the expression of the *Foxo3* gene associated with lifespan (Forkhead box O3) [89]. A deficiency of the enzymes that produce H_2_S leads to hypertension, while the administration of chemical H_2_S donors lowers blood pressure and protects against organ damage in animal studies [90]. MST is a gasotransmitter H2S enzyme implicated in bone mineralization. Its role in prevention of cartilage calcification during development of osteoarthritis has been recently documented [91].

In addition, it was found that oxidation stress, Cys-SSOH) deprivation or inhibition are accompanied by increased expression of CGL (cystathionine γ-lyase gene) resulting in increased H_2_S production staying in negative balance with mTORC1 (related to protein syntheses). The increased amount of H_2_S is a key indicator of the caloric restriction diet effectiveness [92]. Plasma concentration of H_2_S is also capable of modulating inflammatory responses, has antioxidant properties, and regulates the vasodilatory effect [93]. The concentration of H_2_S in blood plasma is gradually reduced with age, therefore, it could be suggested as a biological age marker [85]. The H_2_S donor chemical sodium thiosulfate has already been successfully used in clinical trials for the treatment of calciphylaxis in patients with the terminal stage of renal disease [94]. Nevertheless, due to the side effect of thiosulfates, it is necessary to find new H_2_S donors [90,94]. Maintaining the H_2_S balance in blood may be a promising strategy for developing a geroprotective therapy.

### 2.5. β2-Microglobulin

β2-Microglobulin (β2M) as a part of the major histocompatibility complex (MHC) is present on the surface of platelets, lymphocytes, and monocytes. Expression of β2M is regulated by interferons and pro-inflammatory cytokines, and its function is critical for immune surveillance. It stabilizes MHC to establish antigen presentation. Platelet-derived β2M is a mediator of pro-inflammatory differentiation of monocytes and may play the role of a chaperone in carcinogenesis or as a myocardial protector [95]. Accumulation of β2M takes place in blood plasma as a result of the inflammatory response, but is actively eliminated by renal filtration. The decline of the renal function with age causes a gradual increase of the β2M amount in blood plasma and the progression of age-related impairments of both the cognitive function and the neuro-regenerative processes [95,96,97].

The presence of β2M in blood provokes the formation and deposition of amyloid fibrils (amyloidosis), which is the cause of many pathological conditions. This process may be associated with chronic inflammation and aging and may underlie Alzheimer’s disease, Parkinson’s disease, and type II diabetes [98]. Amyloidosis also occurs after long-term dialysis, because modern systems cannot release large molecules such as β2M. This is why this molecule is recognized as a marker of kidney filtration efficiency [99], predictor of all-cause mortality and other cases of health decline and disease risk [100]. Association between the serum β2M level and frailty of elderly people (slowness, weakness, low physical activity, exhaustion, and shrinkage) was also demonstrated [101,102]. Thus, β2M is considered a predictive marker for several age-related inflammatory diseases.

## 3. Circulating Hormones and Growth Factors Associated with Aging

**Figure 1:**
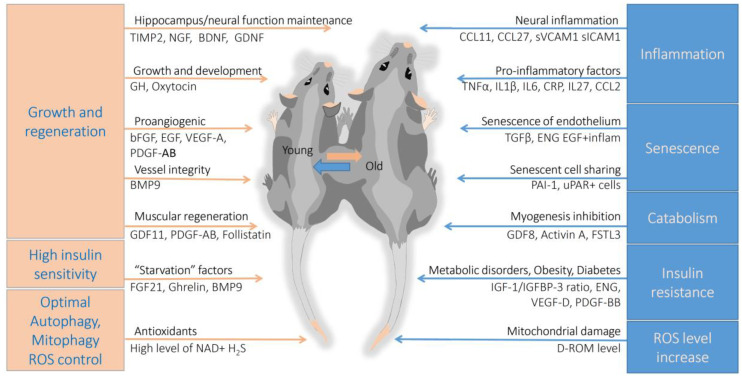
Parabiotic mice model: reciprocal influence of circulation factors on biological aging. In a young organism, the processes of growth and regeneration, high insulin sensitivity, an increased level of cellular cleansing (autophagy, mitophagy) prevail over the processes of accumulation of senescent cells, insulin resistance, catabolism in muscular and other cells, and chronic inflammation (inflammaging) specific for the old organism. Pink arrows indicate factors that are predominantly present in the blood of young animals. The blue arrows show processes and factors–indicators of biological age increase. The parabiotic model with surgically connected vascular system and common blood circulation where factors and cells are exchanged between young and old mice is shown. The model allows assessing the influence of blood factors on biological aging. Several factors shown in the Figure were detected using the parabiotic model (additional description in chapter 3). sVEGF and sICAM1 indicate soluble forms of proteins. “EGF + inflame” shows high levels of EGF signalling combined with high levels of inflammatory factors causing endothelial cell senescence. All names of secreted proteins are explained in the text. We use the names of genes and abbreviations of proteins according to https://www.genecards.org.

The concentration of blood plasma components changes with age. Some of them are indicators of natural aging and at the same time may directly affect, increasing or decreasing, the rate of aging. Parabiotic models, where young and old animals were surgically stitched together, allowed testing how blood plasma components affect health and the rate of aging (Figure 1) [103,104]. The effect of improving health indicators of old animals in parabiotic models was observed in skeletal muscles, heart, liver, and the central nervous system, as well as the simultaneous deterioration of these organs and tissues in young donor mice [105,106,107,108].

Moreover, injection of blood plasma of young mice into the old ones increases the expression of the cyclic AMP response element-binding protein (CREB) in the aging hippocampus. This leads to increased plasticity of neurons and improved cognitive function [109].

A recent study of the expression profile of young and old parabiotic animals identified gene complexes associated with rejuvenation of the vascular system, including improved mitochondrial function and response to oxidative stress [52].

Isolation of active rejuvenation factors from blood plasma is an important strategy, as parabiosis itself is not clinically translatable: young-to-old human blood infusion would be ethically unacceptable and fraught with multiple side effects [110,111,112,113]. Besides, the critical factors functionally linked to aging are excellent biomarkers for biological age estimation. Most remarkable age-related factors are discussed in this chapter (Figure 1, Table 2).

### 3.1. TGF-β Superfamily

The transforming growth factor β (TGF-β) superfamily includes several molecules, such as the bone morphogenetic protein (BMP), growth differentiation factor (GDF), activins, and other. They regulate tissue morphogenesis in the embryo and are involved in several biological processes in adults, such as cell quiescence, apoptosis, differentiation, proliferation, and cell migration, especially playing a pivotal role in the regulation of hematopoiesis. Their abnormal expression is implicated in the pathogenesis of many diseases and aging [114,115,116].

The canonical function of the TGF-β factor is to inhibit growth by transcriptional downregulation of c-Myc (proliferation regulatory factor) and interleukin receptors, as well as by inducing inhibitors of cyclin-dependent kinases that induce cell dormancy. Thus, TGF-β plays a role in many processes as an anti-inflammatory factor. TGF-β expression is elevated during aging. Surprisingly, this rise causes an expansion of pro-inflammatory niche cells in the neurogenic niche hippocampus, in the myogenic niche of skeletal muscles with age. By this manner, TGF-β increases inflammation instead of its canonical role of suppressing immune responses. Blocking of TGF-β signaling by the Alk5 inhibitor reduces the β2-microglobulin level and enhances both neuronal and muscular regeneration in mice [110,117].

Activins bind to specific type II receptors A (ActRIIA) or B (ActRIIB) (for activin A or B, respectively). This binding stimulates phosphorylation of the cytoplasmic proteins Smad2 and Smad3, thereby delivering signal transduction into the nucleus [118]. In addition to the developmental role of activins, they are also associated with inflammation [119].

Other TGF-β superfamily members, growth differentiation factors 8 and 11 (GDF8 or myostatin, and GDF11), along with activins, also transmit signals through SMAD2/3 to the nucleus to regulate a variety of processes: neurogenesis, kidney and endocrine pancreatic development, muscular and heart regeneration. GDF11 protein sequence is 90% identical to GDF8, but they play different roles as ligands for ALK4/ActRII signaling [118,120,121].

Several TGF-β superfamily members, including GDF8 (myostatin), GDF11, and activin A, in blood plasma are recognized as indicators of aging and their putative involvement in the aging process is discussed [118,122]. Besides, GDF8 (myostatin) plays an important role in slowing down the postnatal muscle growth in many animal species. In addition, a mutation in GDF8 leads to improved heart function in aging mice [123] and normalization of fat metabolism [124]. Hence, the increase in its level with age leads to the degradation of both skeletal and smooth muscles [125]. A natural antagonist of GDF8 activins is follistatin. Administration of follistatin intravenously in a muscular atrophy mouse model improves muscular regeneration [126].

The deletion of GDF8 produces a hypermuscular phenotype, while the deletion of GDF11 is lethal in embryos and associated with broad-scale developmental defects, including vascular and muscular atrophy. IGF11 is also involved in vascular cell development and adhesion during neuronal and vascular regeneration in the hippocampus [127,128]. The deletion of GDF11 in skin cells affects the production of dermal matrix components and is associated with skin aging [129]. In line with these studies, reduction of the GDF11 concentration in old adult blood plasma is associated with senile muscular dystrophy [130]. When administered intravenously, GDF11 is capable of reversing age-related cardiac hypertrophy [122,131,132] and protecting against endothelial injury [128]. However, long-term systemic injection of GDF11 can induce cachexia (weakness and body weight loss) [133]. Additionally, a high concentration of GDF11 can lead to a delay in the last stage of erythrocyte maturation and to anemia [134]. Despite conflicting reports on the effects of GDF11 on life expectancy and rejuvenation, blood levels of GDF8 and GDF11 can be used as a predictive biomarker of cardiovascular risk. Decreased plasma amount of GDF11 has also been associated with impaired health, neurodegeneration, and mortality risk [118,122,128].

Recently, activin A has been shown to influence the formation of muscle mass in primates [135,136]. The main receptor for activin A, as well as for GDF8 and GDF11, is the activin type IIB receptor (ACVR2B/ActRII) that transmits signals via SMAD2/3 to the promoters of the key genes responsible for tissue catabolism. One of the ActRII signaling reporters detected in blood plasma is follistatin-like 3 protein (FSTL3). The level of FSTL3 in blood correlates with age [118]. FSTL3 has been proposed as an indicator of accelerated aging of the cardiovascular system and the risk of heart dysfunction. Interestingly, FSTL3 inhibits the ActRII receptor ligands, so the total pools of GDF8 and GDF11 decrease with age; however, the level of activin A increases. Perhaps, activin A is independent of the inhibitory effect of FSTL3. The degradation of muscles and myocardium with age is associated with the increase of the activin A amount in blood plasma [137,138]. Moreover, complete blockade of ActRII protects the myocardium from induced infarction in mice [139]. ActRII signaling has multiple effects not only on the myocardium, but also on blood cells. FSTL3 plays an important role in the production of red blood cells [140] and the adhesion of clonogenic precursors of blood lineages [141], which is possibly associated with age-related changes during hematopoiesis.

The catabolic function of the ActRII receptor depends on the type of ligand that activates this signaling pathway. Another important TGF-β superfamily ligand for ActRII associated with aging is the bone morphogenetic protein (BMP9) [121]. Blockade of BMP9 causes bleeding during development, suggesting its role in maintaining the vascular endothelium structure [142]. BMP9 interacts with ActRII via an additional endothelial specific adapter receptor, endoglin (ENG) [143]. ENG attenuates BMP9 signaling to SMAD3. The reduction in the BMP9 level during metabolic syndrome and cirrhosis was observed along with the increase in the ENG level [144,145]. The growing interest in BMP9 signaling also promotes discussion about the negative role of BMP9/ENG in tissue fibrosis [121]. Additionally, BMP9 is involved in the regulation of lipid homeostasis and glucose metabolism. This hormone can reduce glycogen accumulation in liver. By elevation of insulin synthesis and an increase of tissue insulin sensitivity, BMP9 enforces glucose consumption by muscle tissues and promotes transition of the white adipose tissue to the brown adipose tissue [115]. The big variety of the ActRII signaling pathway inhibitors and their complex post-transcriptional maturation regulation obscure the understanding of their role in aging and their use in testing the biological age [131].

Together, all these studies may suggest monitoring TGFβ, BMP9, GDF11, GDF8, activin A, and FSTL3 as a predictive platform for estimating the age-related compromising health status and rate of aging. 

### 3.2. IGF-1

Insulin-like growth factors (IGFs), including IGF-1 and IGF-2, can bind to the IGF-1 receptor (IGF1R) to regulate cell survival, differentiation, migration, and proliferation at the tissue level, as well as somatic growth, developmental progression, and aging at the body level [146,147]. In plasma, IGF ligands are presented in complexes with IGF-binding proteins (IGFBPs), which block the signaling through the receptor [148].

The blood level of IGF-1 reaches its maximum during adolescence and then gradually declines [149]. At the same time, the increased IGF-1/IGFBP-3 molar ratio in serum of the elderly is positively associated with the all-cause morbidity and mortality, including cancer, diabetes, cardiovascular and cognitive disorders, while the IGFBP-3 level itself is negatively associated with the all-cause mortality [150].

In addition to the main role of the IGF-1/PI3K/AKT/mTOR (Phosphoinositide 3-kinase/ Akt- Protein kinase B /mammalian target of rapamycin) pathway in growth and regeneration, it is also involved in inflammation and cellular senescence. During aging, all tissues, including the immune system, gradually increase insulin resistance diminishing the lymphocytes’ ability to activate and expand in response to infections. This reduces the functional capacity of the immune system [151] and leads to the accumulation of cells damaged by infection, mutated and senescent cells which elude removal by the immune surveillance mechanism [37,152].

On the one hand, depletion of IGF-1 in the circulation can silence IGF-1/PI3K/AKT/mTOR signaling in many animal models and prolong lifespan [153,154]. On the other hand, the IGF-1/PI3K/AKT/mTOR pathway is an important regulator of immune defense, which is also important for lifespan [155,156,157]. Ectopic activation of mTOR shifts lymphocyte polarization towards cytotoxic T cell fate, promoting cell exhaustion, aging, and the accumulation of senescent cells. Mammalian TOR is also involved in the regulation of the inflammatory immune response, increasing the risk of mortality at old age. In the TORC1 complex, mTOR reduces autophagy, which is a major part of intracellular immunity. In line, mTOR inhibitors (geroprotectors, rapamycin analogs (sirolimus, everolimus)) decrease “gerolavic” infection rates in old patients [13] and help to cope with severe viral infections [152]. In addition, an increase in the total level of IGF-1 shifts the naive lymphocyte differentiation towards CD4+ cells which, in combination with active T cells, can lead to the development of an autoimmune response [156,158].

IGF-1/PI3K/AKT/mTOR signaling attenuates an important transcription factor FOXO3A associated with lifespan. Blocking the IGF-1/PI3K/AKT/mTOR signaling pathway leads to an increase in lifespan of various organisms [159,160,161,162]. In addition, a mutation in one of the PI3K domains leads to the activation of FOXO3A [163]. In line with these observations, augmented expression of FOXO3A was found in families of centenarians in Japan and Germany [164]. The well-known inhibitors of IGF-1/PI3K/AKT/mTOR working as geroprotectors, metformin and rapamycin, as mentioned above, modulate immunity and protect against age-related diseases such as diabetes and cancer [13,165,166,167]. Some natural components, for example, propolis, are capable of increasing the expression of lifespan-associated genes, FOXO3A and NGF, and thus also act as geroprotectors [168,169]. Metabolic factors are extremely important for growth, development, and regeneration; however, excessive chronic stimulation of these signaling pathways leads to insulin resistance and promotes cell senescence development.

### 3.3. NGF and Neural Regeneration

Nerve growth factor (NGF) is a neurotrophic factor that regulates the development and maintenance of central cholinergic neurons, sympathetic and sensory peripheral neurons [170]. Nobel prize winner Dr. Rita Levi–Montalcini, who discovered the NGF, applied NGF in the form of eye drops for the rest of her life and lived 103 years [171]. Although the role of NGF in significantly prolonging life expectancy has not been clearly demonstrated, some studies have shown its effect on the suspension of certain senile diseases and prolongation of the functioning of vital body systems [12,42,122,167,172,173,174,175]. Decreased NGF levels are associated with cognitive decline and Alzheimer’s disease. NGF can attenuate the progression of age-related Alzheimer’s disease by decreasing amyloid-β-peptide aggregation in the brain [176]. Numerous clinical trials are attempting to improve the delivery of NGF to neural tissues in the brain to enhance the therapeutic effect of Alzheimer’s disease treatment [177]. A decrease of serum concentrations of other hormones has also been shown in cognitively impaired patients: brain-derived neurotrophic factor (BDNF), glial cell line-derived neurotrophic factor (GDNF) [178].

A potential mechanism for neuronal regeneration can be illustrated by the p85β mutation in the PI3K domain which interrupts the IGF-1/PI3K/AKT/mTOR signal transduction in neuronal precursors. As a result, the lifespan-related transcription factor FOXO3A is dephosphorylated and transferred into the nucleus where it binds to DNA and activates downstream genes, providing strong resistance to oxidative stress, increasing autophagy, and prolonging life [163]. Apparently, PI3K/AKT blockade by p85β mutation or chemical inhibition alters NGF signaling to the MAPK pathway (mitogen-activated protein kinases) and, together with FOXO3A, improves neuronal survival, differentiation, and regeneration [179,180].

NGF is also involved in oocyte development and regeneration through the stimulation of the PI3K/AKT/mTOR signaling pathway [181]. Nasal administration of NGF can dramatically increase serum testosterone concentrations in aging animals. Treatment with NGF has been suggested as a potential therapy for age-related hypogonadism. In a senescence accelerated mouse model (SAMP8) with accelerated aging, NGF increases the activity of hypothalamic neurons and stimulates secretion of the gonadotropin-releasing hormone, which significantly increases sexual motivation and performance, improves sperm quality, and restores fertility of aging males [175].

Thus, plasma NGF levels may serve as an early indicator of Alzheimer’s disease and a promising drug for prolonging the fertile age and combating age-related hypogonadism.

### 3.4. PDGF/VEGF Vascular Remodeling

Platelet-derived growth factors (PDGFs) are composed of several protein chain subunits (A, B, C and D) and regulate vascular metabolism and growth. Heterodimer PDGF-AB level is reduced with age. It has been suggested for the treatment of age-related cardiovascular disorders [182,183]. PDGF-AB has multiple effects on vascular growth, including neovascularization in cancer and inflammation. PDGF-AB is also implied as a factor of bone regeneration [184]. Apical endothelial growth factor VEGF-A (vascular endothelial growth factor, isoform A) is also involved in neovascularization and peripheral nerve regeneration. VEGF expression also reduces with age, which leads to a decline of peripheral vessel regeneration and, consequently, to tissue hypoxia [185]. Mass analysis of 18 million parameters of healthy individuals of various ages and people in the pre-diabetic state revealed a series of factors possibly functionally related to aging and the development of metabolic disorders. Thus, in addition to VEGF and PDGF-AB, they reported that the amount of two other isoforms, VEGF-D (isoform-D) and homodimer PDGF-BB, also increases with age [23]. VEGF-D regulates blood vessel and lymphatic vessel formation and migration and is also involved in the progression of various pathological processes, including pulmonary edema, cancer, inflammation, and obesity. Interfering with this protein level in blood may be a beneficial strategy for research in cardiovascular disorder treatment [186]. However, the role of systemic VEGF-D overproduction in aging is not clear yet.

PDGF-BB expression is indicated in murine fibrosarcoma; it also induces intratumoral lymphangiogenesis and contributes to lymphatic metastasis [187]. The VEGF-D and PDGF-BB level in blood is suggested as a sign of fat tissue accumulation and progressive obesity [188,189]. Perhaps, the cumulative ratio of VEGF-D and PDGF-BB in blood to excess weight indicates the degree of the lymphatic system development within the adipose tissue and shows a healthy trend towards weight loss. PDGF-AB, PDGF-BB, VEGFs, and well-known other factors related to vascular remodeling, bFGF (Basic Fibroblast Growth Factor) and EGF (Epidermal Growth Factor), can be used as indicators of the state of the vascular network and for monitoring age-related pathologies.

### 3.5. FGF21

Caloric restriction (CR) is a well-known dietary therapy that can improve health, prolong life, protect from cardiovascular and metabolic disorders, and reduce the symptoms of neuroinflammatory diseases [7,8,190]. The search for genes upregulated after CR revealed several factors mimicking the CR effect, including transcription factor FOXO3A and soluble hormone FGF21 (fibroblast growth factor 21) [191,192]. CR induces hepatocytes to secrete Fgf21, which inhibits IGF-1/GH1/signaling presumably through SIRT pathway activation [193]. Moreover, overexpression of Fgf21 in mice increases their lifespan [192]. Although FGF21 administration shows promising but moderate improvements in clinical trials of diabetes treatment, the amount of this marker may indicate a healthy response to a high-calorie diet compared to patients with metabolic disorders [194,195]. Elevated blood levels of FGF21 reduce sugar consumption in mammals, while knockout of *Fgf21* gene increases sugar intake in rodents. In humans, the *Fgf21* gene mutation is associated with increased consumption of sweets and alcohol, as well as with daily smoking [196].

### 3.6. Oxytocin

Hormone oxytocin is well-known as the “love hormone” or “cuddle hormone.” It is secreted when a person is in love, cuddling, or is in a friendly social interaction. The hormone injection stimulates muscle regeneration, activation of muscle stem cell proliferation in aged mice via MAPK/ERK (mitogen-activated protein kinases/ extracellular signal-regulated kinases) signaling pathway activation [197]. Administration of the TGFβ/ALK5 signaling inhibitor in combination with oxytocin in old mice downregulates cyclin-dependent kinase (CDK) inhibitor p16 (marker of cellular senescence) and re-establishes tissue regeneration [110]. The response of the organism to this hormone once again demonstrates the importance of care and attention to elderly people.

### 3.7. Growth Hormone

Growth hormone (GH or somatotropin) reaches its maximum level in adolescence, guiding growth and development. GH concentration correlates with IGF-1 levels. GH is involved in the fine regulation of insulin and IGF-1 signaling, but does not affect IGF-2. Adults with the mutations in the *GHRHR* (growth hormone-releasing hormone receptor), or in the *GH1* gene, or with defective downstream signaling (*STAT5B* mutations) have growth retardation and metabolic obstructions [198]. The overpresentation of GH, insulin, IGF-1, or IGF-2 signaling is associated with severe metabolic diseases, overgrowth, and obesity [146].

The level of circulating GH after rising in the young age gradually decreases with age in various species, including humans [199,200,201]. However, mutations in *GH1* also enhance mouse sensitivity to insulin. In the liver of “dwarf” mice (*GH1*-mutated), the insulin receptor, insulin receptor substrates (*IRS-1* and *IRS-2*), are upregulated [202]. At the same time, the systemic insulin level is reduced in these mice, which also increases tissue insulin sensitivity and the animal’s lifespan [190,203]. Moreover, in human families with attenuated GH level, the lifespan is increased without significant changes of IGF-1 and IGFBP-3 amount in blood (other factors related to lifespan are discussed above) [204]. However, in adolescence, during intensive growth and development, the concentration of GH is maximal [200]. A recent small clinical trial showed that treatment of patients by recombinant human GH in combination with two anti-diabetogenic drugs used to prevent hyperinsulinemia (dehydroepiandrosterone and metformin) produces rejuvenation effect which can be measured by biological age indicators and by DNA methylation changes (epigenetic age). The thymus regeneration and improvement of other immunological parameters in the treated patients were also observed in this study. As a result, age estimated by DNA epigenetic markers regressed by 1.5 years after one year of treatment [14]. Notably, due to the side effects of therapy, the GH treatment of healthy individuals for the purposes of aging delay or rejuvenation is considered unethical and illegal in the United States [205].

So called “hunger hormone” ghrelin is an endogenous ligand for the growth hormone secretagogue receptor (GHS-R) secreted by the gastrointestinal tract in response to diet restrictions. GHS-R signaling stimulates GH release, food intake, and carbohydrate and lipid metabolism. Besides, ghrelin regulates gastrointestinal secretion, immune function and plays a role in neuronal and cardiovascular cell protection from apoptosis [205].

## 4. Age-Associated Inflammatory Factors

The concentration of inflammatory factors in blood increases with age, which is often referred to as “inflammaging” [11]. This process is associated with the accumulation of senescent cells and with chronic infections often developing at old age [206]. As shown in numerous studies, inflammaging contributes to diabetes [207], skeletal aging [208], inflammatory bowel disease, arthritis, nerve tissue inflammation, chronic lung inflammation, and other terminal illnesses, such as cancer, Alzheimer’s disease, and multiple sclerosis [209,210]. Blocking of inflammation with a TNFα antagonist during rheumatoid arthritis increases sensitivity to insulin, indicating a link between inflammation and metabolic disorders [211]. Moreover, an increase of COVID-19 mortality in the elderly is associated with the level of chronic inflammation in patients [212]. The all-cause mortality in old adults correlates with oral health. Inflammation of gum, oral mucosa, periodontium is associated with the risk of stroke, myocardial infarction, rheumatoid arthritis, and cardiovascular disorders and mortality [57]. With the optimal state of immune system at young age, the inflammatory response calms down rapidly after the pathogen is removed from the body. Damaged cells are quickly removed, and healthy tissues are completely restored. With age, the immune response to the pathogen is more and more likely to become chronic, causing sepsis and turning into an uncontrolled process. Older adults also accumulate aging immune cells, which also increases inflammation. [13,51,152,213,214].

### 4.1. Well-Known Pro-Inflammatory Factors

During inflammaging, as the most typical pattern of immune aging, the concentration of tumor necrosis factor alpha (TNFα), interleukins (IL1b, IL6), C-reactive protein (CRP) increases in plasma [11,206,207]. The amount of Neopterin in blood plasma is an excellent marker indicating the chronic inflammation level. This metabolite is secreted by macrophages. It accumulates during physiological aging and autoimmune disorders [215]. Fewer reports describe accumulation of other age-related inflammatory factors, such as soluble TNF receptor-1 (TNFR-1), Monocyte chemoattractant protein 1 (MCP1) also known as CCL2, Vascular Endothelial Growth Factor A (VEGF-A), Epidermal Growth Factor (EGF), Cyclooxygenase-2 (COX-2), Nitric Oxide Synthase, Inducible (iNOS/*Nos2*) [113,216]. Recently, several important factors—potential indicators of age-related inflammation—were reported. However, an increase in the inflammatory factor concentration is also observed for a short time during acute infectious diseases. Therefore, when assessing the biological age using inflammaging markers, the general state of human health should be taken into account, since the presence of an acute infection can drastically change the picture of inflammatory aging assessment and lead to unreasonable conclusions about the biological age of the organism.

### 4.2. CCL2

CCL2 (C-C motif chemokine ligand 2, MCP-1) recruits peripheral monocytes to tissues and supports takeover of the M1 (inflammatory) phenotype inducing inflammation during the innate immune response [217]. CCL2 expression is induced by pro-inflammatory factors such as TNFα, IFNγ, IL-1, IL-4, IL-6, LPS, TGFβ, etc. [218]. CCL2 is referred to as a potential diagnostic biomarker of prostate [219] and breast cancer [220] and indicator of bone remodeling in bone-related metastatic cancers [221]. CCL2 is expressed by a variety of cell types, including endothelial cells, myeloid cells, epithelial cells, astrocytes, neurons, and glial cells and promotes glial activation [218].

CCL2 is significantly upregulated in Alzheimer’s disease. Moreover, CCL2 overexpression via adenovirus delivery in both cortex and hippocampus induces expression of inflammatory factors and pathological Tau protein aggregation in neurofibrillary tangles. Authors hypothesized the key role for CCL2 in Alzheimer’s disease pathogenesis [222]. CCL2 level in blood plasma is also associated with severity of liver disease [217] and significantly elevated in elderly humans with aortic valve stenosis [223,224]. Age-related increase of CCL2 was even more accelerated in *Ercc1-/Δ* and *Bubr1H/H* mouse progeria models. Thus, CCL2 is probably related to the “inflammaging” phenotype; therefore, it has been suggested as a marker of biological age [225].

### 4.3. CCL11

C-C motif chemokine ligand 11 (CCL11), also known as eotaxin, was identified as a pro-aging factor in blood plasma. CCL11 role in aging was discovered in experiments with parabiotic mouse models. The CCL11 level increases with age in blood and cerebral fluid. When this factor was injected into young mice, deterioration in memorization and decline of neurogenesis were observed [226]. CCL11 also tilts the immune response towards Th2 reaction and is involved in recruitment of eosinophils and neutrophils into inflamed tissues. The elevated blood level of this chemokine signifies evidence of an ongoing chronic inflammatory process in the organism. CCL11 is also involved in allergic reactions and asthma progression. It has been suggested as a prognostic biomarker and a candidate for development of future antiaging drug strategies [227,228].

### 4.4. CCL27

C-C motif chemokine ligand 27 (CCL27) plasma concentration is increased in aged adults [23]. This chemokine attracts T cells to the site of inflammation. High level of CCL27 was detected in the saliva of patients with periodontal disease [229]. Remarkably, this illness is associated with the risk of developing age-related cardiovascular disorders [57]. Besides, CCL27 is involved in autoimmunity, cancer, and hypoxia response [230,231].

### 4.5. IL27, IL35, and TNF Interaction

Some circulating pro-inflammatory factors can cause aging of hematopoietic stem cells (HSCs) that support the production of all immune cells in the body. For example, TNFα upregulates IL27Ra via the ERK/ETS1/NF-kB pathway. As a result of IL27/IL27Ra signaling, HSCs acquire the aged phenotype comprising myeloid bias and reduced self-renewal. It has been shown that the level of IL27 in blood is possibly an important marker for assessing the rate of HSC aging [232]. An increase in the IL27 concentration also leads to stress myelopoiesis resulting in the development of abdominal aortic lesions [233]. IL27 levels have been shown to be minimal in early childhood, but the peak of cytokine production by dendritic cells occurs during adulthood and possibly at old age [234]. IL27 ligand is associated with its heterodimeric partner product of *Ebi3* gene (EBV-induced gene 3). Besides, IL27Ra is also ligated with the IL35/Ebi3 complex [235]. Thus, monitoring blood levels of such factors as IL27, Il35, and TNF will stimulate the development of new approaches to examining the blood stem cell aging.

### 4.6. Soluble VCAM1 and ICAM1

Expression of vascular adhesion molecule 1 (VCAM-1) on the inflamed endothelium is upregulated. VCAM-1 can transmit signals to inflamed endothelial brain cells from plasma in old mice and might partly be the reason of age-related neurovegetative disorders. ADAM17 metalloprotease cleaves VCAM-1, thus blocking the inflammatory loop. The increase of the soluble VCAM-1 (sVCAM-1) concentration in plasma is the sign of endothelial activation, inflammation and of health decline in aged adults [47].

Intercellular adhesion molecule 1 (ICAM-1) promotes lymphocyte intravasation into inflamed tissues. Its expression is upregulated on endothelial cells. It is also cleaved during anti-inflammatory loop activation. Age-related accumulation of the soluble form of ICAM-1 (sICAM-1) in blood plasma is an indicator of health decline and frailty in old adults [236].

The renin–angiotensin system (RAS) plays a key role in regulating blood pressure, electrolyte balance, and vascular tone. It also induces angiogenesis by activating the synthesis of VEGF-A, bFGF, and can modulate the inflammatory response by enhancing the synthesis of metalloproteases and the expression of sICAM-1 and sVCAM-1. Long-term presence of both molecules in blood plasma is a signal of local inflammation and a sign of metastasis in cancer [24,237,238].

### 4.7. vWF

The von Willebrand factor (vWF) was discovered in a study of inherited bleeding disorders. It is involved in the early stages of clot formation. When platelets gather at the site of bleeding, vWF acts like glue, helping them clump together to stop blood loss [239]. Likewise, for any tissue injury or inflammation, vWF supports the recruitment of platelets and leukocytes to inflamed and damaged foci [240,241]. In all these pathological conditions, vWF synthesis is enhanced and, accordingly, its presence in the circulation increases [240]. An increase in the level of the vWF protein in blood is an indicator and one of the causes of chronic endothelial inflammation and the risk of immunothrombosis. This molecule modulates activation of the endothelial surface and tissue permeability for the intravasation of lymphocytes to the site of inflammation [240,241,242].

During aging, the vWF level rises following inflammaging and accumulation of senescent cells with an inflamed phenotype [241]. An increase in the level of the vWF protein in blood is an indicator and one of the causes of chronic endothelial inflammation and the risk of immunothrombosis. This molecule modulates activation of the endothelial surface and tissue permeability for the intravasation of lymphocytes to the site of inflammation [240,242]. During aging, the vWF level rises following inflammaging and accumulation of senescent cells with an inflamed phenotype [241].

It is also involved in atherosclerotic plaque formation by activating the vascular surface [243] and considered a predictive biomarker for age-related disorders, including diabetes, stroke, myocardial infarction, and sepsis [240]. Thus, plasma vWF level is an excellent early indicator of chronic endothelial tissue inflammation for biological aging research.

### 4.8. TIMP2-Anti-Inflammaging

Tissue inhibitors of metalloproteinases (TIMPs) are known to be endogenous inhibitors of matrix metalloproteinases. The plasma of the umbilical cord blood of newborns contains large amounts of TIMP2 [26,244]. Moreover, experiments on parabiotic animal models showed that a systemic TIMP2 pool is required to maintain normal hippocampal function and improve cognitive function of older animals [26,109,245]. TIMP2 is constitutively expressed in microglia, but is significantly inhibited during inflammation [245]. TIMP2 plasma level decreases in both humans and mice with age [244]. The effect of the dung beetle glycosaminoglycan known as an anti-aging and anti-cancer substance was related to upregulation of TIMP2 and the glycosaminoglycan’s anti-inflammatory activity [246]. However, another study reports *TIMP2* correlation with cancer progression [247].

The anti-inflammatory anti-aging effect of TIMP2 suggests that small molecules or other intrinsic metalloproteinase inhibitors may decelerate age-related inflammation.

### 4.9. Soluble uPAR and PAI-1

The accumulation of senescent cells is one of the main processes causing aging and the occurrence of chronic age-related diseases. Recently, it has been shown that clearance of senescent glial cells prevents neurodegenerative processes, cognitive dysfunction, and age-related disorders in mice [248,249]. Inhibition of the FOXO4 interaction with p53 induces apoptosis in senescent cells and restores tissue homeostasis, fitness, fur density, and renal function in aging mice [250]. Moreover, cell senescence can be stimulated by inflammatory conditions and, vice versa, senescent cells secrete pro-inflammatory cytokines and induce a feedback loop of senescence [31,251]. Transplanting senescent cells into young animals causes physical dysfunction and accelerates aging of the hosts [41]. Thus, monitoring the pool of senescent cells will allow determining the biological age of an individual with greater physiological accuracy.

The plasminogen activator system consists of the urokinase-type plasminogen activator (uPA) and its receptor (uPAR). Serine protease inhibitors (serpins), including plasminogen activator inhibitor-1 and -2 (PAI-1 and PAI-2), contribute to fibrinolysis, cell adhesion and migration, but also play an essential role in cell senescence and tumor development. PAI-1 secretion by senescent cells has been recently reported [252]. Moreover, blocking PAI-1 by chemical inhibitor TM5441 reduces age-related cellular senescence in cardiac myocytes, endothelial cells, and fibroblasts [253].

Endogenous protein kallistatin prevents vascular injury. It can promote telomerase activity, inhibit oxidative stress, and suppress endothelial progenitor cell TNFα-induced senescence by reducing expression of PAI-1 [254].

Recent research revealed uPAR is also expressed on the surface of senescent cells [255]. The plasma concentration of soluble uPAR correlates with inflammation and accelerated biological aging. Individuals with high levels of soluble uPAR have a greater decline in cognitive function and physical activity [256]. Thus, soluble uPAR and PAI-1 could be excellent biomarkers of biological aging.

## 5. Conclusions

The aging of an organism is accompanied by an increase in its biological age, which, in theory, should correlate in time with the calendar age. However, some internal pathological conditions and environmental influences can slow down or accelerate the process of natural aging [2,4,6,9,10,257,258].

### 5.1. Vascular and Neural System Aging

Under the pressure of inherited and environmental factors, the density of the capillary network per unit of volume reduces with age. This is associated with an increase in body weight and with a natural decrease in the expression of growth factors that regulate vascular growth, including EGF, VEGF, bFGF, PDGF-AB, BMP9/ENG (Table 2) [54,145]. A decline in the number of neurons regulating the vasomotor response of vessels due to a decrease in expression of NGF and other factors leads to a reduction of the maximum lumen size of capillaries and weakens the response to stress. Along with hormonal changes, the attenuation of the renin–angiotensin system [24,238,259,260], impaired growth and regulation of the vasculature also cause a decline in muscle activity, an increase in blood clotting (also due to the high vWF level), and a rise in blood pressure with age [260]. In addition, a decrease in hemoglobin concentration in erythrocytes results in hypoxic disorders in peripheral tissues, which in turn increases the likelihood of pathological disorders in the organism. A general weakening of the immune response, aging of immune cells, and diminished blood supply to tissues cause chronic foci of infection. This, in turn, changes biochemical blood plasma indicators, in particular, enhances the secretion of inflammation indicators, such as PUFA, neopterin, β2-microglobulin, fibrinogen (Table 1).

### 5.2. Systemic Inflammaging

At the same time, in blood plasma, there is the increase in pro-inflammatory cytokines (IL1β, IL6, IL27, TNFα), chemokines (CCL11, CCL27), and the molecules involved in lymphocyte trafficking to the place of inflammation (soluble VCAM1, ICAM1, and vWF) (Table 2) [226].

Another sign of age-related inflammation is the accumulation of uPAR+ senescence cells, which secrete pro-inflammatory cytokines PAI-1 and TGFβ. The latter slows down the proliferative activity of immune cells and can also cause the conversion of niche cells to a pro-inflammatory phenotype [117]. The increase in the concentration of inflammatory markers in blood plasma is also associated with the damage to the neural and cardiovascular systems and can significantly reduce life expectancy per se [206,210]. Age-related accumulation of metabolites in blood can increase the inflammatory response, which also often leads to the cardiovascular system injury and renal dysfunction (Table 1) [97,101,112,117]. The albumin or BUN/creatinine ratio and elevated calcium level in blood are associated with premature death risk and reduced lifespan [58,59,210].

### 5.3. Regeneration and Metabolic Disorders

During aging, inflammation, metabolic changes, as well as decreased regeneration and repair cause development of senile diseases. Age-related changes in the expression of factors affecting muscle tissue regeneration (e.g., GDF11, PDGF-AB, etc.) and an increase in catabolic factors (GDF8, activin A, etc.) lead to a slowdown of heart and skeletal muscle regeneration in response to injury [118,127,133].

A decrease in growth hormone and oxytocin levels with age, on the one hand, is associated with a general slowdown in growth and development of all body systems, including regeneration processes. On the other hand, due to an increase of the IGF-1/IGFBP-3 ratio, this stimulates the predominance of high mTOR-dependent metabolic activity and insulin resistance [150].

In a healthy cell, mitochondria produce superoxide dismutase (SOD), which neutralizes reactive oxygen species (ROS), protecting cellular organelles from damage. High activity of the mTOR complex reduces autophagy, mitophagy, and SOD production. As a result, the number of damaged mitochondria increases, which in turn stimulates the accumulation of ROS, contributing to further cell damage [29,33,37]. Together, increased metabolic activity, impaired mitochondrial function, insufficient concentration of protective redox molecules (e.g., H_2_S), and inflammation prompt high spending of NAD+ and ROS accumulation [85,86,261]. These events of cellular aging further trigger advanced pathological processes, accumulation of senescent cells, and development of age-related disorders [262].

### 5.4. Perspectives

Blocking factors that negatively affect lifespan is a reasonable strategy to prevent early disability and prolong the active life of older people. Among such strategies, tested in the clinical practice or which will be translated to the clinical practice, one can highlight overcoming the insulin resistance by diet restriction [8], increasing FGF21 in blood circulation, pharmacological treatment of insulin resistance (e.g., with dehydroepiandrosterone and metformin) [12,14], stimulation of tissue repair by GH, oxytocin, GDF11 and TIMP2 [203], vascular regeneration with bFGF, EGF, VEGF, PDGF-AB, and BMP9, preventing the development of “inflammaging” by administering anti-inflammatory molecules, including COX-2 inhibitors, leukotriene receptor antagonists, TIMP2, or other matrix metalloproteinase inhibitors [210,263], overcoming the cell senescence by administration of TM5441 analogs, optimizing the autophagy and mitophagy with mTOR inhibitors (rapamycin analogs) [264], with TGF-β inhibitors [110,264], antioxidant therapy [265,266], reduction of NAD+ exhaustion [73]. The indicators and mechanisms discussed above reflect the natural and pathological aging processes. In this review, we propose a comprehensive monitoring of indicators and markers of biological age, showing functional changes in an aging organism (Table 1 and Table 2). We also come close to the promising avenues for further research to develop health protection, aging delay, and rejuvenation prospects for older people to support healthy aging and prolong active life.

## Figures and Tables

**Table 1:** Vascular conditions, metabolites, lipids, and redox agents as risk factors of age-related disorders and their influence on lifespan.

Circulating Indicators of Aging	Dynamics during Aging	Function/Risk Factor in the Elderly	Reasons of the Condition	Lifespan Influence
Age-dependent vascular disorders				
Capillary network	Decrease	Reduction of the peripheral tissue oxygen level	Reduction of VEGF-A and PDGF-AB level	Negative
Maximum capillary diameter	Decrease	Thrombosis, high blood pressure	Angiotensin system disorder, neural atrophy	Negative
Number of sensor neurons	Decrease	Stress-dependent blood pressure	Neural function decline, VEGF level reduction	Negative
Inflammation indicators				
Fibrinogen	Increase	Tissue inflammation	Immunity misbalance, autoimmunity	Negative
PUFA	Increase	Tissue inflammation	Immunity misbalance, autoimmunity	Negative
β2-Microglobulin	Increase	Inflammation	Renal function decline	Negative
Neopterin	Increase	Inflammation, autoimmunity	Intracellular infections	Unknown
Anti-inflammatory indicator				
Sphingomyelin	Decrease	Cell protection, intestinal infection protection	Age-related decline of sphingomyelin synthase	Positive
Kidney disorder indicators				
BUN/creatinine ratio	Increase	Risk of cardiovascular mortality	Renal function decline	Negative
Albumin/creatinine ratio	Increase	Risk of cardiovascular mortality	Renal function decline	Negative
Calcium	Increase and level variation	Risk of cardiovascular and cancer mortality	Renal function decline	Negative
Lipids				
Abundancy of different classes of lipids	Decrease	Vascular protection/nutrition	Lipid metabolism disorders	Unknown
Reactive oxygen species/antioxidants				
NAD+	Decrease	Supports energy processes, antioxidant	Inflammation, metabolic misbalance	Positive
ROS (D-ROM/TTL ratio)	Increase	Risk of cardiovascular, diabetes, and cancer mortality	Aging process, *Nrf2* level decline	Negative
H_2_S	Decrease	Regulation of 1nflammation, antioxidant	CGL oxidation stress response decline	Positive

**Table 2:** Dynamics of growth factors, hormones, and inflammatory factors involved in aging.

Circulating Molecule	Dynamics during Aging	Function/Risk Factor in Elderly	Molecule Longevity Influence
Growth factors and hormones variation during aging			
TGF-β	Increase	Niche and nerve cells senescence induction	Negative
GDF8	Increase	Muscular regeneration block, obesity, heart failure risk	Negative
GDF11	Decrease	Muscular regeneration, improved angiogenesis in the brain	Positive
Activin A	Increase	Cardiovascular and heart dysfunction risk	Negative
FSTL3	Increase	Cardiovascular and heart dysfunction indicator	Unknown
BMP9	Decrease	Endothelial maturation and integrity, new vessel formation inhibition, insulin resistance inhibition. A high level of ENG is a sign of diabetes or liver disease.	Unknown
IGF-1/IGFBP-3	Increase	All-cause mortality risk, reducing autophagy, insulin resistance	Conditional
NGF, BDNF, GDNF	Decrease	Neurons survival, prevention of cognitive decline	Positive
PDGF-AB	Decrease	Cardiovascular and heart dysfunction risk reduction	Positive
VEGF	Decrease	Vessel growth and remodeling	Conditional
VEGF-D and PDGF-BB	Increase	Lymphatic vessel formation in fat tissue, insulin resistance	Unknown
FGF21	Decrease	Starvation factor, IGF/GHR signaling suppression, Cell survival under stress support	Positive
Oxytocin	Decrease	Muscle and tissue regeneration	Positive
Growth hormone (GH)	Decrease	Muscle and tissue regeneration, insulin resistance rise	Conditional
Ghrelin “hunger hormone”	Decrease	Secreted in response to diet restriction, regulates GH secretion and energy metabolism. Impact on obesity and insulin resistance.	Conditional
Pro-inflammatory factors			
TNFα, IL1β, IL6, CRP	Increase	Chronic increase indicates tissue inflammation and age-dependent immunity dysfunction	Negative
CCL2	Increase	Aortic inflammation, Aortic stenosis	Negative
CCL11	Increase	Recruitment of immune cells to inflamed tissues, cognitive function decline	Negative
CCL27	Increase	Recruitment of T cells to inflamed tissues, cognitive function decline	Negative
IL27	Increase	Blood stem cell aging, tissue inflammation, infection, stress response, vascular damage	Negative
Soluble VCAM1 ICAM1	Increase	Indicator of immune cells recruitment to inflamed tissues	Negative
vWF	Increase	Vascular inflammation, risk of diabetes, stroke, myocardial infarction	Negative
Anti-inflammatory factors			
TIMP2	Decrease	Hippocampus function maintenance	Positive
Senescence indicators			
PAI-1	Increase	Immune cells senescence indicator	Negative
Soluble uPAR	Increase	Immune cells senescence indicator	Negative
Kallistatin	Unknown	Oxidative stress inhibition, senescence prevention	Positive
